# Gleanings

**Published:** 1894-07

**Authors:** F. H. Lutze

**Affiliations:** Brooklyn, N. Y.


### GLEANINGS.

F. H. Lutze, M. D., Brooklyn, N. Y.

### Generalities.

Ants running through body; sensation of. Cistus.

Aneurism. Baryta-mur.

Bones; periosteum of; drawing, tearing, worse at night; wet weather or stormy, and at rest; better from motion. Rhod.

Child wants to be carried only by its mother, but no one else; screams when touched. Anti-tart. (Cina, Cham.)

— — — — — — — — — — but if mother gets weary she can rock it instead. Cina.

— — — — — — fast. Ars., Brom.

— — — — — — slow. Puls.

— is affectionate and yawns all the time; screams when awaking. Ignat.

— — peevish and irritable and starts on touch. Kali-c.

Eruption on face better from cold applications. Apis, Alumina, Puls., Psor., Sulph.

Cold, takes easy; knows not how. Tuberc.

Convulsions come on suddenly, without warning, or are preceded by jumping or starting in sleep. Bell.

— after rage. Cham.

— recur often; opisthotonos; pale face, and dark rings around the eyes; cerebral anæmia. Cicuta.

Cracking in joints. Caps., Ledum, Nitr-acid., Petrol.

Descending aggr. Bell., Borax, Stan.

Empty; worse when stomach is empty. Anacard., Lach., Phos.

Epilepsy; aura in solar plexus; a creeping sensation up through chest or stomach. Silicea, Bufo.

Emaciation in little children. Marum.

Jerking hiccough after nursing, in children; belching without bringing up anything; crying. Marum.

Discharges from ear, nose, anus, or vagina; with fish-brine odor.

— from rectum, etc. Calc., Carb., Medorr.

— — ulcer —. Graph.

— — vagina —. Sanicula.

— — ears. Tellur.

Flushes of heat at climacteric. Cimicif., Coccul., Lachesis, Sang., Sep., Veratr-vir., Ambr-gris., Puls., Sulph., Oleum-an., 1.30 P. M.

Fright aggr. Arnica flower tea, Cupr., Hyoscyam.

Ganglion. Calc-c.

Fanned, wants to be, hard. Apis, Carb-v.

— — — — gently. China.

— — — —. Cistus, Plumb., Puls., Sulph.

Measles, after undeveloped, or badly treated fevers. Carb-v., Psorin.

Hammer, as if struck suddenly with, on right side of head, when walking anywhere, out-doors or in, which always throws him to the left. Tabac.

Growing pains (so-called, are really rheumatic pains), with weakness or fatigue. China.

— — with hyperæsthesia, no exhaustion. Cimicifug.

Incurables to relieve. Ars., Rhus-t., Lachesis, Tarentula.

Hydroa, white or pearly and more singly. Natr-m.

— clearer tendency to yellow or amber color, and more in clusters. Rhus-tox.

Jarring aggravates pain in head. Bell., Glon., Spig.

— — — — stomach. Sepia.

— region of liver is sensitive to a jar. Natr-sulph.

Jarring, aggr. headache in back of neck going up right side of head, or left, pains sharp, darting at times, throbbing in temples. Gossypium.

— head sensitive to, or rattling of wagon or stepping hard. Nitr-ac., Sulph.

— aggravates pains in abdomen and uterus. Lil-tig.

— aggravates sitting in chair or lying in bed. Aloe.

— headache from. China.

— sensitive to and worse from jarring the bed. Bell.

Music ameliorates, must be sung to sleep. Tarent-hisp.

— aggravates. Natr-sulph.

Noise aggravates. Alum., Borax., Caust., Chin., Merc.

Open air, a constant irresistible desire to walk in open air. It does not fatigue. Fluor-ac.

Paralysis of both legs and right arm. Cann-ind.

— progressive upward. Con.

— from exhaustion of nerve power in infectious diseases. Kali-phos.

— on trying to walk there is an inclination to run forward. Mangan.

— of right arm and left leg. Ars., Tereb.

— Cicut., Kali-phos., Latyrus, Gels., Mangan, Physostig. Sil., Psor.

Pains go to side lain on. Bry., Puls.

Rheumatism from wet cold, worse at rest, better from warmth, left shoulder and right hip. Nux-m.

Baby seizes hold of nurse when being carried, from dizziness. Gels.

— — — — — — — lowered in bed. Borax.

— — — — — for fear of being separated. Cuprum.

Sleep, aggravation on falling to. Amm-c., Carb-v., Grindel-robust.

— on going to, respiration ceases. Amm-c., Anti-tart., Badiaga, Carb-an., Carb-v., Digit., Graph., Grindel-rob., Lachesis, Op., Ran-bulb.

— when baby is put to, will sleep twenty to thirty seconds, then wakes with a start and screams. Lachesis.

Sour smell from whole person. Iris-v., Hyper., Magnes-c., Rheum, Sulph-acid.

Stiffness of old age; worse A. M., takes a long time to limber up. Phos.

Sycosis. Ars., Anti-cr., Aur-mur., Baryt., Clematis, Natr-sulph., Sarsap.

Touch, worse from. Argent-met., Merc-cor.

— sensitive to on throat. Bell., Kali-bichro., Lachesis.

Restlessness of feet, and effects from getting feet wet. Calc-c.

— — — — — — getting head wet. Bell.

Syphilitic affections of bones.

— — — pinkish red swelling of tibia nodes, with unbearable pain. Kali-iod.

— ulcers of roof of mouth. Aurum.

— single small tumors of roof on mouth, with suppuration. The tumors are discolored and the bone deeply involved in suppuration. Asafœt.

— nodes, bluish on tibia. Mangan-acet.

— tibia small and numerous, affecting the periosteum. Asafœt.

In children, when Ars., Calc., and Silicea fail, then frequently *Æthusa* will help.

Burns, trismus after extensive. Amyl-nitros.

Air, warm, cannot endure, must have doors and windows open. Aml-nitros.

Fanned, has to be, must have fresh air, it seems he cannot survive for want of fresh air. Apis, Cann-ind., Carbo-veg., China, Cistus., Plumb., Puls., Sulph.

Journey, when about to make; fear, trembling, restlessness, prostration, cold sweat. Ars.

Covered, desire to be. Calc-c. (Secale-cor.).

Lying with head low, horizontally, relieves headache. Calc-c.

— — — raised relieves headache. China.

Mestastasis of mumps to testes; suppressed mumps or exanthemata; gangrene, with burning in stomach; no thirst, acidity. Carbo-veg.

Cool room, working in, ameliorates, aggravation in heat of sun. Fluor-ac.

Warm room, working in, ameliorates, much worse in cold air. Silicea.

If some one spot in body will not yield though other symptoms of disease have been cured, and other symptoms correspond. Fluor-ac.

Draught, noise, sensitive to least. Hep-s-c.

Sour, whole person smells sour. Iris-v., Hyper., Magn-c., Rheum, Sulph-ac.

Suffocation and choking worse from touch. Lachesis.

Congestion to head, worse from eating and exertion. Naja.

Craving for ice, amorous dreams. Elaps.

Complaints return regularly every fourteen days, but not at same hour. Lachesis.

— — — — fourth day at the same hour. Sabadilla.

Throat, chest, and ovarian affections begin on left side and go to right; rheumatism begins on right side and goes to left. Lachesis.

Flushes of heat, followed by profuse warm perspiration. Sulph.

— — — — — — — — — which soon becomes cold. Lachesis.

— — — associated with mental depression, as if a cloud had settled over the patient; distressed and suspicious without cause. Cimicifug.

Lying on right side, causes a pulling soreness in left chest and all complaints worse A. M. Natr-c.

Paralysis, painless (in left upper parts, spasmodic contraction). Oleand.

Lying on left or painless side aggravates. Phos.

Chilliness, thirstlessness, and oppression of chest. Puls.

Position must change, better after it for a few moments, better from rapid motion. Rhus-t.

— — — better from slow motion. Puls.

— — get up and move about slowly to get relief, he cannot sit still. Magnes-c.

Rheumatism worse from slightest motion, yet was forced to move the leg; must get a new position, but there was no relief. Puls.

— cannot sit still, must move constantly. Rhodod.

Children smell sour, also stool, breath, vomit. Rheum.

Complaints resulting from sudden and thorough drenching by a shower of rain, or getting wet in any way; aversion to washing. Rhus-tox.

Bones painful, injuries to periosteum. Ruta-grav.

— tender to touch; inflammation of bones; caries; bones bend. Silicea.

Grasping automatically of hands to head, nose, throat, ears. Stram.

Aggravation in general of Valerina toward evening, from being still; great sleeplessness in early part of night. Valer.

Pains darting from within outward. Valer.

Chorea, with cold perspiration, could not lift one foot without the other. Veratr-vir.

Convulsions, eyes wide open, but insensible to light, staring look, pupils dilated, eyes turned to the left, mouth closed, teeth clenched, froth oozing from between teeth and lips, face pallid, drawn to the left, convulsions confined mostly to the left side, the right remains passive; opisthotonos. Cicuta-vir.

— preceded by sudden blindness. Cuprum.

— head drawn firmly back with rigidity of muscles of posterior neck from use of tobacco. Lycopod.

Epilepsy, the aura begins in the knees and ascends till it reaches the hypogastric region, then unconsciousness, foam at the mouth, falling down convulsed. Cuprum-acetic.

Bones, diseases of:

Inflammation, chronic. Mercur.

— due to mercurialization. Aur., Nitric-acid, Staph.

— due to syphilis. Merc., Kali-iod.

Caries. Fluor-acid, Silicea, Phos., Phos-acid; when there is hectic and free suppuration, then give for sequestrum detachment of, Symphit.

Osteitis. Merc., Mezer., Acid-Nitr., Acid-Phos., Phos., Staph., Aur., Sil., Calc., Hep., Iod., Kali-bichrom.

— in tubercular diathesis. Phos., Calc., Natr-m., Silicea, Iod., Sulph.

Caries of temporal teeth. Kreos.

### Euthanasia.

Phos.$^{cm}$ (one dose only), hectic fever in full blast; skin hot in afternoon; night sweat; constant burning thirst; red spot on cheek; diarrhœa; stool escapes on coughing; intense fever in afternoon; constriction of chest, suffocation (an aggravation will follow, but it will soon pass off, leaving the patient free of fever and more comfortable, etc.).

Lachesis, suffocation, distress in chest and stomach, streaming perspiration, great sinking, must have neck free of clothing, also chest and abdomen; ghastly countenance. (Give the 200 or higher as often as necessary.)

Carbo-veg., covered with cold sweat, must be fanned; abdomen is distended with flatus, breath cold. Give Carbo-veg. in aq. every hour for six hours, then stop.

Ars., Secale, death pains, dying cells, mortification in abdomen.

Tarent-cub.$^{30}$, much later in the last stage of consumption it helps to throw the mucus out.

Child cries all day, especially from four to eight or nine P. M., *draws knees up to abdomen; relieved somewhat by lying or pressing on abdomen*, but sleeps good all night; straining at stool, the stool is hard and not often (every second or third day). Colocynth.

